# The Impact of the Pandemic on Acute Ischaemic Stroke Endovascular Treatment from a Multidisciplinary Perspective: A Nonsystematic Review

**DOI:** 10.3390/ijerph18189464

**Published:** 2021-09-08

**Authors:** Milda Grigonyte, Agne Kraujelyte, Elija Januskeviciute, Giedrius Semys, Oresta Kriukelyte, Egle Kontrimaviciute, Nomeda Rima Valeviciene, Dalius Jatuzis

**Affiliations:** 1 Faculty of Medicine, Vilnius University, 01513 Vilnius, Lithuania; milda.grigonyte@mf.stud.vu.lt (M.G.); kraujelyte.agne@gmail.com (A.K.); elija.januskeviciute@mf.stud.vu.lt (E.J.); giedrius.semys@gmail.com (G.S.); 2 Clinic of Anaesthesiology and Intensive Care, Institute of Clinical Medicine, Faculty of Medicine, Vilnius University, 01513 Vilnius, Lithuania; Egle.Kontrimaviciute@santa.lt; 3 Department of Radiology, Nuclear Medicine and Medical Physics, Institute of Biomedical Sciences, Faculty of Medicine, Vilnius University, 01513 Vilnius, Lithuania; Nomeda.Valeviciene@santa.lt; 4 Clinic of Neurology and Neurosurgery, Institute of Clinical Medicine, Faculty of Medicine, Vilnius University, 01513 Vilnius, Lithuania; Dalius.Jatuzis@santa.lt

**Keywords:** acute ischaemic stroke, emergency, anaesthesia, airway management, mechanical thrombectomy, SARS-CoV-2, COVID-19

## Abstract

Background: At the beginning of the coronavirus disease 2019 (COVID-19) pandemic, reduced admissions for cerebrovascular events were identified, but acute ischaemic stroke (AIS) has remained one of the leading causes of death and disability for many years. The aim of this article is to review current literature data for multidisciplinary team (MDT) coordination, rational management of resources and facilities, ensuring timely medical care for large vessel occlusion (LVO) AIS patients requiring endovascular treatment during the pandemic. Methods: A detailed literature search was performed in Google Scholar and PubMed databases using these keywords and their combinations: acute ischaemic stroke, emergency, anaesthesia, airway management, mechanical thrombectomy, endovascular treatment, severe acute respiratory syndrome coronavirus 2 (SARS-CoV-2), COVID-19. Published studies and guidelines from inception to April 2021 were screened. The following nonsystematic review is based on a comprehensive literature search of available data, wherein 59 were chosen for detailed analysis. Results: The pandemic has an impact on every aspect of AIS care, including prethrombectomy, intraprocedural and post-thrombectomy issues. Main challenges include institutional preparedness, increased number of AIS patients with multiorgan involvement, different work coordination principles and considerations about preferred anaesthetic technique. Care of these patients is led by MDT and nonoperating room anaesthesia (NORA) principles are applied. Conclusions: Adequate management of AIS patients requiring mechanical thrombectomy during the pandemic is of paramount importance to maximise the benefit of the endovascular procedure. MDT work and familiarity with NORA principles decrease the negative impact of the disease on the clinical outcomes for AIS patients.

## 1. Introduction

The global coronavirus disease 2019 (COVID-19) pandemic was declared by the World Health Organization (WHO) on 11 March 2020. Despite the fact that at the beginning of the pandemic, reduced admissions for cerebrovascular events were identified, acute ischaemic stroke (AIS) remains one of the leading causes of death and long-term disability [1,2]. The reduction in stroke admission rates may be the result of increased difficulty in accessing medical services, reduced medical provision of scheduled services, late admissions or even patients’ personal fears of visiting the hospital during the pandemic [3,4]. Reported decreases of stroke patients range from 30% to 60%, similar to reductions seen in other acute conditions (e.g., gastrointestinal haemorrhages and myocardial infarctions) [4]. Although the exact cause behind these trends has not yet been established, it seems unlikely to be caused by a decrease in the incidence of these conditions; it may, instead, be a result of social distancing decreasing early identification of diseases or patient’s fears about coming to the hospital in the midst of a pandemic [4]. Due to complex pathophysiology, COVID-19 disease may manifest with diverse presentations and complications [5]. Although severe acute respiratory syndrome coronavirus 2 (SARS-CoV-2) mainly affects the respiratory system, there is evidence that damage occurs throughout the body, including the neurological system [5].

Globally, large vessel occlusion (LVO) AIS care is driven by a multidisciplinary team (MDT). An increasing number of studies are being performed to improve the understanding of how these stroke care teams work and manage AIS patients. The aim of this article is to review current literature data and present the newest knowledge for MDT coordination, rational management of resources and facilities, ensuring timely high-quality medical care for LVO AIS patients requiring endovascular treatment during the pandemic.

## 2. Materials and Methods

A detailed literature search was performed in Google Scholar and PubMed databases using these keywords and their combinations in medical subject headings terms: acute ischaemic stroke, emergency, anaesthesia, airway management, mechanical thrombectomy, endovascular treatment, SARS-CoV-2, COVID-19. Published studies and guidelines from inception to April 2021 were screened. The results from the primary literature search were reviewed, and suitable articles were retained. Bibliographies were reviewed for any studies that may have been missed by the primary literature search. Non-English articles were not included. The focus will be on adult patients with AIS requiring endovascular treatment during the pandemic, since a limited number of articles review and formulate medical service optimisation for patients demanding urgent multidisciplinary medical help. The following nonsystematic review is based on a comprehensive literature search of available data, wherein 59 of the most relevant articles reflecting AIS patients care changes during the pandemic were chosen for detailed analysis. Because of the heterogeneity of studies (i.e., pathology-related, different anaesthetic management approaches, clinical outcome, etc.) and the expected lack of information for many of the treatment stages included, advanced statistical techniques such as meta-analysis were not performed.

## 3. Results

A Review of Literature.

### 3.1. Incidence

AIS remains one of the leading causes of death and long-term disability [1,2]. The COVID-19 pandemic has had a negative impact on certain aspects of stroke care quality provision to non-COVID-19-related AIS patients, as well as to those who suffered a stroke as a complication of COVID-19 disease [6].

#### 3.1.1. AIS in Non-COVID-19 Patients

A study that included 25 hospitals in New England found that the rates of stroke admissions decreased during the pandemic and noted a general reduction in various stroke quality of care measures, though the timelines of stroke care delivery were not affected [6]. This may be the result of public concern and the diversion of emergency department (ED) resources to COVID-19 patients. A prospective study in an academic stroke centre with a significant urban catchment population conducted during the COVID-19 lockdown in Vilnius, Lithuania, similarly found a decreased rate of stroke alerts and admissions, as well as a decrease in prehospital stroke triage quality and significant hospital arrival delays from symptom onset, but the timeliness of stroke care delivery in the ED was not negatively affected by the lockdown [7]. It is difficult to ascertain the exact reasons behind the successful preservation of in-hospital stroke care goals during the COVID-19 pandemic. This is likely to be due to administrative efforts made to reorganise the ED to maintain effective functioning during the pandemic, as well as the in-hospital prioritisation of stroke patients due to their generally critical condition and the need for time-sensitive treatment [6].

#### 3.1.2. AIS in COVID-19 Patients

AIS can be one of the presenting symptoms of SARS-CoV-2 infection, and current evidence suggests that COVID-19 is capable of damaging the central nervous system and causing various neurological complications, including stroke [8,9,10]. The incidence of AIS in COVID-19 patients is a particular area of interest, as prothrombotic states marked by increased D-dimer concentrations have been reported in hospitalised COVID-19 patients, compounded by public reluctance to seek emergency health care in the COVID-19 era, owing to fears of contracting the infection, which may lead to undesirable delays in administering time-sensitive stroke treatment [11,12,13,14]. Despite current data showing reduced rates of stroke admissions during the pandemic, it is important to note that the full extent of stroke incidence in COVID-19 patients is difficult to accurately gauge, as evaluating critically ill, intubated and heavily sedated patients for acute neurological deficiencies is difficult, time-consuming and not routinely performed; incidentally, this group of patients may be at highest risk of developing AIS as a complication of SARS-CoV-2 infection, some of which may ultimately remain undiagnosed due to death from the underlying illness. A systematic review by Tan et al. reported incidences of AIS among COVID-19 patients in five hospitals in different countries ranging from 0.9% to 2.7%, with an overall pooled incidence of 1.2%, with greater rates of stroke in patients hospitalised with a severe form of the infection [15]. A study by Merkler et al. conducted in two New York hospitals found that the rate of stroke was higher in patients hospitalised or attending the ED due to COVID-19 infection compared to patients presenting with influenza; the authors did not, however, compare the incidence of AIS in COVID-19 patients to the general population [16]. A study in Wuhan found that stroke occurred in 5.7% of critically ill COVID-19 patients compared to 0.8% of those with milder forms of the disease; a later meta-analysis reported an overall stroke incidence of 1.1% among COVID-19 patients, a figure that is up to 3 times higher than in the general hospital population [17].

### 3.2. Pathogenesis of COVID-19-Related Stroke

AIS is a heterogeneous syndrome caused by a disruption in the perfusion of the brain by thrombotic or embolic conditions, which presents with a wide variety of focal neurological deficits that correlate with the area of the brain that is affected [18,19,20,21]. It is thought that AIS may occur in up to 1.1% of all COVID-19 cases [17].

COVID-19 infection is being increasingly recognised as an independent risk factor for AIS, affecting even young and otherwise largely healthy patients who would not normally be considered to be at risk of stroke [8,9,10,22]. Multiple pathophysiological mechanisms for ischaemic stroke in COVID-19 patients have been proposed, but the exact underlying mechanism has yet to be determined [22]. Instead, it is helpful to look at stroke in COVID-19 patients as the result of the interaction of several underlying mechanisms [23]. Increased D-dimer concentrations among COVID-19 patients experiencing AIS suggest activation of the coagulation and innate immune system, and those with more severe forms of COVID-19 disease tend to have greater D-dimer concentrations [17,21]. The high incidence of stroke in COVID-19 patients may be partially explained by the tendency for critically ill COVID-19 patients to be older and have more underlying diseases, making them more at risk for stroke; however, it is unlikely that this alone can fully explain the high incidence of stroke in COVID-19 patients [24]. Additionally, the occurrence of cytokine storms has been associated with critically ill COVID-19 patients, resulting in elevated levels of interleukin 6 and C-reactive protein; both have been associated with increased risk of stroke [17,22]. SARS-CoV-2 affinity for angiotensin-converting enzyme 2 receptors may also play a part by directly causing vasculitis in the brain vascular endothelium as a result of viral uptake of these receptors, as well as by impairing cerebral vascular autoregulation [22,25]. Additionally, COVID-19 disease may contribute to an increased risk of stroke by decreasing oxygen supply to the brain as a result of severe pulmonary infection [22].

### 3.3. Prethrombectomy Issues

#### 3.3.1. Screening, Prehospital Care and Triage at the ED

Avoiding delays in LVO recanalisation is crucial for achieving optimal outcomes, and AIS patients could benefit from mechanical thrombectomy within 24 h from the “last seen normal” time [26]. Considering the urgent nature of acute stroke and the time frame required to rule out COVID-19, routine use of disease transmission preventive measures such as hand washing, social distancing and personal protective equipment (PPE) during the initial assessment is recommended. If feasible, it is worth considering telemedicine, as it has been shown to be effective in remotely assessing the National Institutes of Health Stroke Scale (NIHSS) score, as well as eligibility for mechanical thrombectomy. A direct transfer to a larger hospital should be considered [27]. For inpatient stroke codes, clinical information can be obtained from the patient’s chart and nursing staff prior to assessing the patient in person. The patient is considered COVID-19 positive until proven otherwise, both on arrival at the treatment facility and with an inpatient stroke code [28].

During the COVID-19 pandemic, a new challenge in triaging stroke patients emerged. Interfacility transfer, which occurs in 44–72% of AIS patients, requires special equipment and personnel, and these arrangements are likely to delay the time to procedure [29].

Urgent testing is performed when the patient arrives at the ED: the rapid bedside COVID-19 test and any other routine laboratory tests. If there is a patient with positive pulmonary symptoms, it is recommended to consider the need for chest computed tomography (CT) simultaneously with head and neck CT/CTA [28]. Current guidelines for acute stroke management recommend that all suspected AIS patients undergo brain CT scan on arrival within a 20 min time frame; contrast angiography is required for those being considered for mechanical thrombectomy [30].

It is important to follow protocols (WHO, Centers for Disease Control and Prevention, local) when managing AIS patients, including guidelines for hand washing, PPE use, COVID-19 testing and self-quarantine if needed, as well as to organise a simulation training for staff [27,31]. Optimisation of AIS triage at the ED requires a multidisciplinary approach and collaboration between many different providers of stroke care [31].

As acute stroke care is time critical, it is important to administer the appropriate treatment regardless of COVID-19 status. In COVID-19 patients requiring acute stroke care, the duration of successful recanalisation has been shown to be prolonged by up to 25 min [28]. Furthermore, functional outcomes after endovascular procedures are reported to have worsened during the pandemic [32]. The American Heart Association and the American Stroke Association suggest following the 2019 Update to the 2018 Guidelines for the Early Management of AIS in the treatment of COVID-19 patients experiencing AIS [31]. In addition to the above recommendations, it is temporarily proposed to minimise the number of emergency medical personnel present during the examination and treatment of infected AIS. During the pandemic, there is a need for more active use of telemedicine [31,33]. Televideo consultations are proven to be superior to telephone consultations [34].

#### 3.3.2. Interventional Radiology (IR) Suite and Room Set-Up

It is necessary to ensure the provision of appropriate IR services, including endovascular treatment of AIS during the pandemic. Developing an IR room and procedural organisation plan to provide safe and effective endovascular services may aid in achieving this goal. Consideration should be given to redesigning every aspect of the IR suite in response to the COVID-19 pandemic, including the path of transfer, laundry and medical waste removal.

IR patients are divided into three categories: (1) confirmed COVID-19; (2) infection status not yet determined; (3) uninfected patients that tested negative for the infection. Confirmed COVID-19 cases are transferred to a negative-pressure IR suite, whereas patients without exclusion are transferred to a quarantined IR suite and patients without COVID-19 to a regular IR suite. The three different categories involve different levels of preventative and protective measures [35]. Elevator use and minimal duration of stay in the recovery suite are recommended when moving infected patients [36]. It is appropriate to perform interventional procedures according to pre-established schedules in order to avoid the mixing of infected and noninfected patients, to adapt interventional suite design to minimise different room exposures and to ensure that waiting periods in the preparation room are kept to a minimum [36,37]. The IR suite should contain only vital equipment; all other equipment can be brought in from the preparation suite by staff when required [37]. All procedural elements in the suite should be prepared before the arrival of the patient (medications, devices, etc.) in order to minimise the amount of time that the patient spends inside the suite, protect equipment from possible contamination and to avoid breaking scrub. It is worth considering partially or completely isolating the IR department floor where CT scans are performed. To this end, the recovery area can be turned into a PPE donning and removal area, thus providing direct and convenient access to the interventional suite. Mujoomdar et al. suggested appropriate isolation room design with staff movement suggestions before and during procedure [38]. Personnel who are on the IR service premises but not directly involved in the procedures are advised to stay at least two metres away from the patient and away from the patient’s movement path [36]. Adequate ventilation in the IR room that is similar to the operating room should be ensured. The best solution to ensure personnel protection and reduce the likelihood of viral spread is to use high-efficiency particulate air (HEPA) filtration systems in the interventional suites while creating negative pressure in the suite. One of the possible solutions is to install a pressure monitor in the room to ensure a pressure differential of −2.5 Pa has been reached. When feasible, the same approach can be applied to waiting rooms, bathrooms and decontamination rooms. If emergency intubation is required during the procedure, all staff should leave the room except those directly involved in the procedure [28]. After the interventional procedure, contaminated PPE is discarded in the adjacent small room to avoid viral spread, followed by cleaning and disinfection of the patient’s transfer path [36,38]. In cases of confirmed COVID-19 patients or when infection status has not yet been determined, it is vital to clearly indicate that a procedure is being performed on a patient with COVID-19 on the entrance to the IR suite (for example: DO NOT ENTER) [39].

### 3.4. Intraprocedural Issues

#### 3.4.1. Stroke Team Emergency Preparedness and Multidisciplinary Approach

For confirmed AIS with LVO, rapid mechanical thrombectomy is the standard of care. Adequate response to the pandemic and hospital staff emergency preparedness are crucial in order to ensure high-quality AIS care.

In general, the number of health care professionals involved in the patient’s care should be as low as possible (ED specialists, neurologists, anaesthetists, interventional radiologists, nurses and medical radiation technologists, along with essential staff such as receptionists and cleaning staff) whenever possible during the process of the patient care, keeping at least 1 m distance between [40]. Plan of incorporating the trainee exposure and limiting the number of contacts for academic centres is likely to be developed in accordance with the University policy. During the procedure, it is advised to minimise the number of healthcare workers present in the IR suite to the operator, nurse and a single anaesthetist attending, all wearing appropriate PPE [41].

Pandemic response and preparedness for AIS stroke care bring attention to several chronic weaknesses of interventional neuroradiology and nonoperating room anaesthesia (NORA). Hospital staff members with different clinical backgrounds involved in the patient’s management operate as a team, but teamwork brings new challenges during the pandemic. Therefore, it is crucial to expect a more difficult working environment due anti-infective precautions, as well as due to mechanical thrombectomy intraprocedural workflow issues related to process (lack of communication during urgent procedures, interventional radiology staff is not familiar with anaesthesia principles), difficult settings (limited patient access due to C arm, monitoring beyond the X-ray shield, the environment is noisy), management of procedural-related emergencies (different types of bleeding (sudden vs. delayed onset) due to vessel perforation, haematoma at access point and non-CNS complications such as contrast reactions or contrast nephropathy), and recognise the need of multidisciplinary approach implementation [42,43]. More positively, researchers from various countries (Switzerland, Canada, USA) and societies globally are starting to recognise the importance of stroke team preparation and present new directions for MDT coordination [24,32,44].

#### 3.4.2. What the Neurologist Needs to Know

Mechanical thrombectomy is an invasive treatment method for AIS that involves introducing a balloon-guided catheter into the arterial system. Table 1 lists the main indications and contraindications for mechanical thrombectomy [45].

During the pandemic, health care professionals and facilities must be innovative. The need for new initiatives for acute neurological care workflow is the perspective of the nearest future. Telemedicine involvement could be helpful in acute neurology, as well as it could help to prevent from overtriage due to surplus neurologic patients intertransfer [46]. Nevertheless, interhospital transfer poses a risk to infectious disease spread; on the other hand, introduction of qualifying criteria for urgent need for thrombectomy will focus patients who absolutely need a stroke centre [46,47]. It would be useful to require CT angiograms pretransfer to a thrombectomy centre in order to ensure the presence of LVO [48]. Only confirmed cases would be transferred, which could reduce patient flow and lower the risk of in-hospital transmission of COVID-19. Moreover, these changes are relevant even in non-pandemic periods and could play a key role in cost savings and workflow optimisation. Adapting teleneurology may play a more prominent role during the pandemic as it allows for efficient and risk-free evaluation of the patient. However, teleneurology is unlikely to be able to replace physical examinations for accurately diagnosing AIS when the patient presents with subtle symptoms that may be hard to evaluate during a digital consultation [49].

It is important to clarify that one of the most important points is the criteria that specialists decide to adopt for a specific endovascular treatment. Recent scientific data suggest a shift from a “time is brain” to a more physiological “tissue is brain” is recommended [50]. This new concept leads to the wide application of neuroimaging techniques to help the decision process and extending the time window for treatment [51,52]. Advanced neuroimaging has a particularly important implication during the pandemic. Possible treatment delays due to the current crisis related to increased time intervals “onset to door” and/or “door to needle”; therefore, selection of patients for mechanical thrombectomy poses new challenges and requires refinement of established protocols [3,6,7]. Although minimising time to treatment is critical, the decision to perform mechanical thrombectomy should be individualised because current data shows that the association between endovascular reperfusion and improved functional outcome is not strictly time dependent [28,50]. New evidence suggests that some patients who arrived beyond classical time reference and normally would be excluded by the endovascular reperfusion treatment may benefit from thrombectomy, probably in part due to variations in collateral circulation among individual patients [51]. Imaging data are becoming the centre of treatment decisions, and Rehani et al. reviewed the advanced imaging algorithm for AIS workup, describing advanced CT or magnetic resonance imaging (MRI) perfusion imaging (CTP/MRP) techniques that could help to identify patients who would benefit from thrombectomy, and specified a simple decision algorithm addressing when to perform endovascular therapy by imaging criteria and time of stroke onset [50]. Decision for treatment with endovascular thrombectomy is complex and should be based on vascular and physiologic information rather than based on selection according to rigid time windows.

The importance of public education about stroke symptoms cannot be overestimated during the pandemic. As stroke treatment is time sensitive, it is important to devote time and resources to reach out to members of the public who may be reluctant to seek medical assistance due to fear of contracting SARS-CoV-2 infection in the hospital [53].

#### 3.4.3. What the Interventional Radiologist Needs to Know

AIS is a highly time-dependent condition, and the rapid initiation of treatment remains vital in the pandemic.

Time from onset to hospital arrival, hospital arrival to groin puncture time, time from groin puncture to first recanalisation attempt, time from hospital arrival to first recanalisation attempt and time from onset to first recanalisation attempt play a huge role in successful treatment of LVO and are known to be indicators of successful reperfusion. Though the rate of successful reperfusion does not differ a lot in prepandemic and pandemic patients, hospital arrival to puncture time and hospital arrival to reperfusion time differ significantly, mainly reflecting the delay caused by the COVID-19 screening process [54].

Given that neuroradiological findings may be among the first evidence of COVID-19 observed, the interventional radiologist has a critical role to play in detecting and referring for further treatment of strokes, ensuring the safety of downstream personnel and the hospital. Interventional radiologists need to be aware of the neuroradiologic manifestations of COVID-19 [55].

Natural language processing approaches can help automatically track acute or subacute ischaemic stroke numbers for epidemiologic studies, though local classifier training is important due to radiologist reporting style differences [56].

#### 3.4.4. What the Anaesthesiologist Needs to Know

During the pandemic, NORA for AIS care brings more challenges to safe anaesthesia care, but MDT work can facilitate safe and efficient procedural care in the IR suite [57,58].

At some institutions, anaesthetists do not routinely participate in mechanical thrombectomy [44,59]. Therefore, it is recommended that such hospitals consider involving anaesthetists in mechanical thrombectomy during the pandemic, as emergent intubation may be associated with higher risk of exposure for all personnel in the IR suite [44,59]. At the beginning of the pandemic, given that the majority of patients present for mechanical thrombectomy with unknown COVID-19 status, the Society for Neuroscience in Anesthesiology and Critical Care (SNACC), The Society of NeuroInterventional Surgery (SNIS) and European Society of minimally invasive neurological therapy (ESMINT) recommended a lower threshold for intubation, especially if the anaesthetist or interventional neuroradiologist have any concerns for possible conversion from monitored anaesthesia care (MAC) to general anaesthesia (GA) [32]. During this time, researchers tried to identify the impact of the pandemic on LVO management. Tabibian et al. found a decrease in functional outcomes and hypothesised that the routine use of GA for all patients undergoing mechanical thrombectomy during the pandemic may be partially responsible [32]. Although it is important to conduct additional studies to determine the predictors of poor outcomes under these unique pandemic conditions, there are increasing data to consider and prefer conscious sedation as the first choice if the patient is stable [44,59]. Early and controlled intubation with a high-efficiency particulate air (HEPA) filter directly on the tracheal tube is preferred in a patient who is considered at risk for airway deterioration, unable to protect the airway, actively vomiting, agitated or uncooperative [44,59]. It is not recommended to delay extubation unless there is neurological or respiratory deterioration [44,59].

Surgical masks should be worn by the patient while undergoing MAC with as low as possible oxygen flow through the nasal cannula to achieve arterial oxygen saturation > 94% [44]. In addition, anaesthesia staff should carefully consider conversion to GA if the patient continues to remain hypoxaemic. It is advised for anaesthetists to use the pharmacological agents for MAC with which they are most familiar in this setting and to be prepared to safely convert to GA if needed [44]. Conversion to GA may be required due to changes in patient or procedural conditions. Videolaryngoscopy is the safest choice for rapid sequence intubation and is performed by the most experienced airway specialist available; additionally, preventive measures for airborne transmission should be utilised [44]. It is important to minimise any delays in cerebral reperfusion as a result of change in practice, specifically due to the use of GA, while accounting for essential pandemic precautions. As the preparation for intubation in a patient with known or suspected COVID-19 is likely to take longer than regular intubation, it is critical that hemodynamic parameters be strictly maintained in the recommended range while awaiting intubation [44]. To mitigate potential worsening of stroke symptoms due to hypotension, we recommend using an intubation strategy that preserves cerebral perfusion pressure using either etomidate or ketamine, with continuous monitoring of blood pressure. If hypotension does occur, early use of sympathomimetics and a fluid-conservative resuscitation strategy in accordance with Critical Care Medicine COVID-19 Surviving Sepsis Guidelines to maintain systolic blood pressure > 140 mmHg is recommended [44,59].

Understanding the safe practice and the risks associated with NORA for AIS anaesthetic management in IR settings could result in better MDT work coordination.

### 3.5. Post-Thrombectomy Issues

The Anaesthesia Patient Safety Foundation recommends that suspected or confirmed COVID-19 patients should not be extubated within the IR suite but rather in a negative-pressure environment once criteria for extubation are met and not be brought back to existing general postanaesthesia care units. Patients in whom COVID-19 has been excluded, including nonintubated and stable patients, should receive postoperative care according to institutional guidelines or can be moved to a step-down unit with appropriate nursing expertise in the setting of a shortage of critical care beds. When the patient is handed off to the receiving team, the gowned (if needed) provider checks the patient’s neurological exam, vital signs. This can count as the 15–30 min check postprocedure, every hour for two hours and then in a 4 h interval. The frequency of combined neurological, vital signs and access site checks can be adjusted depending on patient status, the patient’s haemodynamic stability and concern for access site bleeding.

A minimum 30 min delay is recommended before perioperative cleaning staff cleans the IR suite to allow the room to air out [41].

## 4. Strengths and Limitations of the Review

This article reviews current available scientific literature and summarises academic and clinical knowledge on working as an MDT involved in immediate AIS care, with a focus on mechanical thrombectomy.

A limitation of this article is that due to the heterogeneity of the available studies and outcomes we reviewed, a fully systematic review methodology was not possible.

## 5. Conclusions

Adequate management of AIS patients requiring mechanical thrombectomy during the pandemic is of paramount importance to avoid the unnecessary time delay and maximise the benefit of the endovascular procedure ensuring the highest-quality care for the most vulnerable patients in the ED and/or thrombectomy room. In the thrombectomy centre, MDT work and familiarity with NORA principles decrease the negative impact of the disease on the clinical outcomes for AIS patients. In preparation for potential future pandemics, and in the interest of infection control in general, it is preferable to have negative-pressure angiography rooms and/or separate areas for anaesthetic induction and postmechanical thrombectomy recovery within the interventional radiology theatres until the result of the COVID-19 test will be known.

The need for more evidence on how COVID-19 causes stroke remains, and this statement pertains to new data and can change as new facts arise.

## Figures and Tables

**Table 1:** Indications and contraindications for mechanical thrombectomy [45].

Indications	Contraindications
Time from symptom onset to arterial puncture ≤6 h	Thrombocytopenia < 30 × 10^9^; known bleeding diathesis; current use of anticoagulant with INR > 3
Patient age ≥ 18 years	Uncontrolled arterial hypertension: systolic blood pressure > 185 mmHg or diastolic blood pressure > 110 mmHg before treatment; blood glucose < 2.8 mmol/L; >22 mmol/L; haemodialysis or peritoneal dialysis
The patient was independent in daily activities before stroke onset (mRS 0–2)	History of intravascular haemorrhage; subacute bacterial endocarditis; severe comorbidities with poor prognosis
ASPECTS ≥ 6 points	The area of acute ischaemia covers ≥ 1/3 of the middle cerebral artery supply area on cerebral CT/MRI; and/or marked mass effect with midline displacement, changes in brainstem or cerebral hemispheres
Mechanical thrombectomy should be considered in patients with LVO within 6–24 h of symptom onset if the results of neurological examination and clinical evaluation are in accordance with the current stroke treatment protocol of the medical institution and allow to expect a favourable prognosis	Evidence of intracerebral or subarachnoid haemorrhage on cerebral CT; evidence of intracranial process with a high risk of bleeding (tumour, abscess, vascular malformation, aneurysm); intracranial surgery or brain injury in the last 3 months

## Data Availability

Data presented in this review manuscript are available in the quoted references.
